# Supplementary material for: Effects of Salinity and Temperature on the Flexibility and Function of a Polyextremophilic Enzyme
Source: Int J Mol Sci. 2022 Dec 9;23(24):15620. doi: 10.3390/ijms232415620 (PMC9779221; doi:10.3390/ijms232415620)
Supplement: Supplementary file 1 [file ijms-23-15620-s001.zip › ijms-2020360-supplementary.pdf]

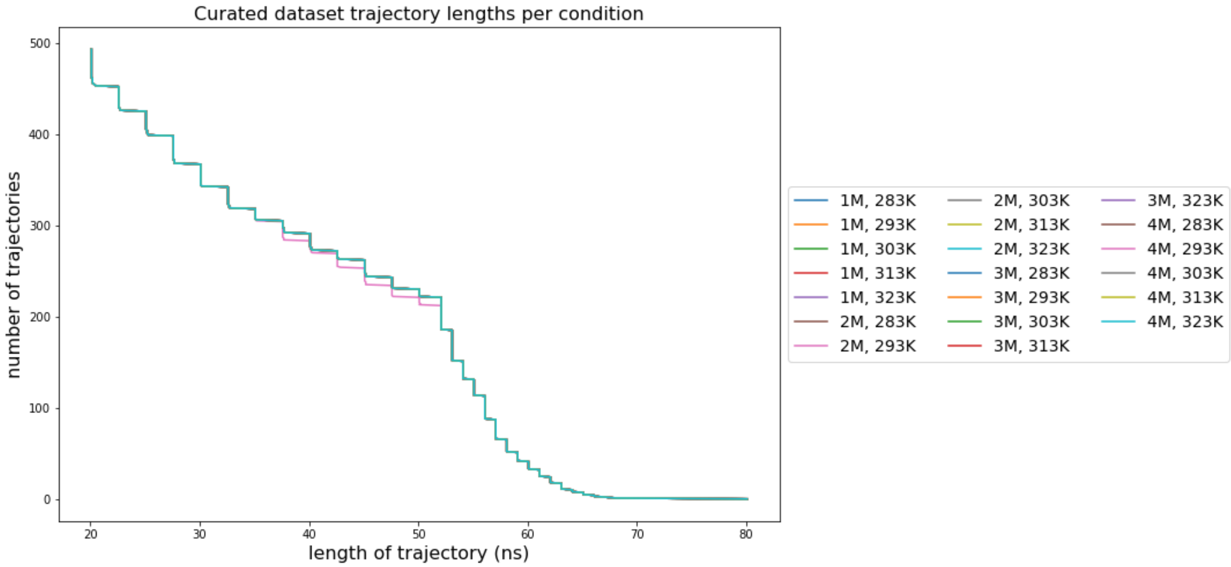

Figure S1. Distribution of trajectory lengths for each condition in our curated dataset. There are 20 conditions, each condition containing 494 trajectories and about 21.1 μs of data. The aggregate simulation time is 422 μs.

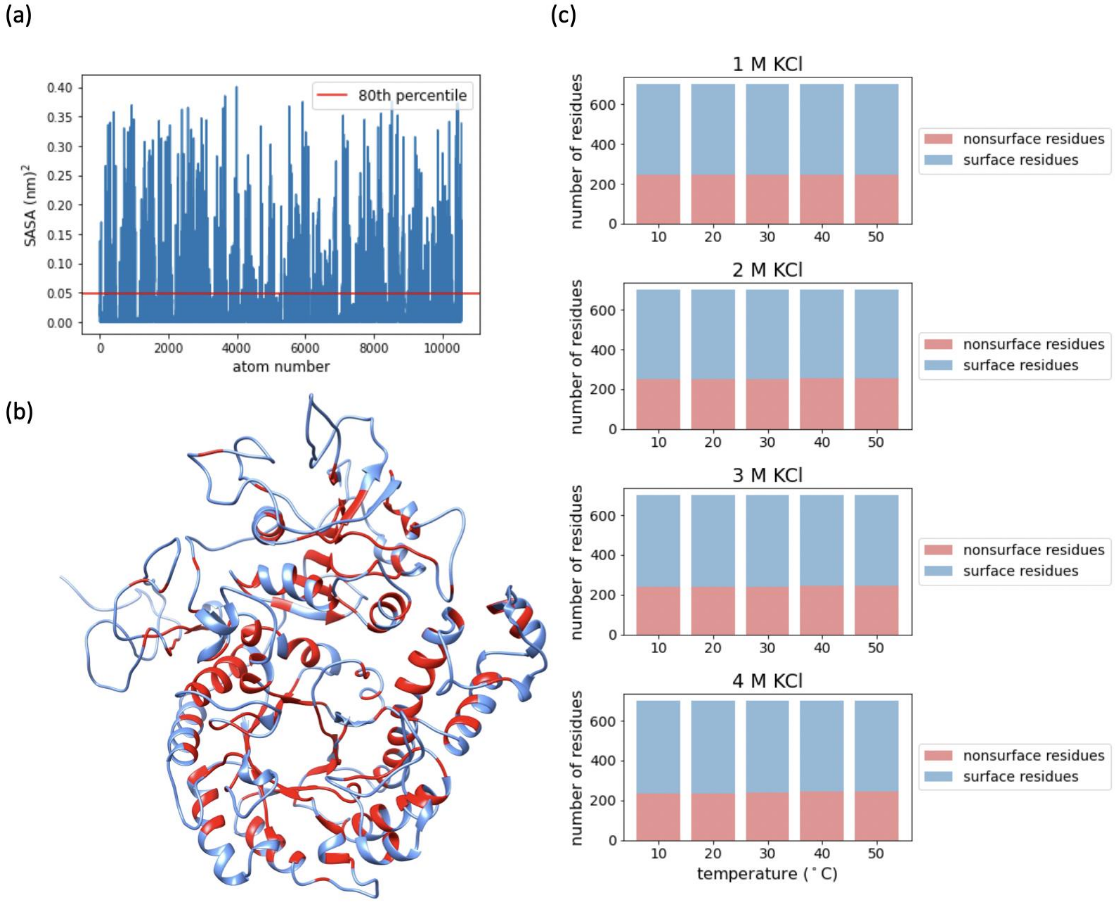

Figure S2. SASA per atom profile for the condition 1M 10 °C, with a red line to show the 80th percentile of these values which acts as the cutoff value to distinguish a residue as a surface residue vs nonsurface residue (a), the surface (blue) and nonsurface (red) residues for the condition 1M 10 °C mapped on the protein, using the 80th percentile value as the cutoff (b), and the number of residues characterized as a surface or nonsurface residue for each condition using the 80th percentile value as the cutoff value (c).

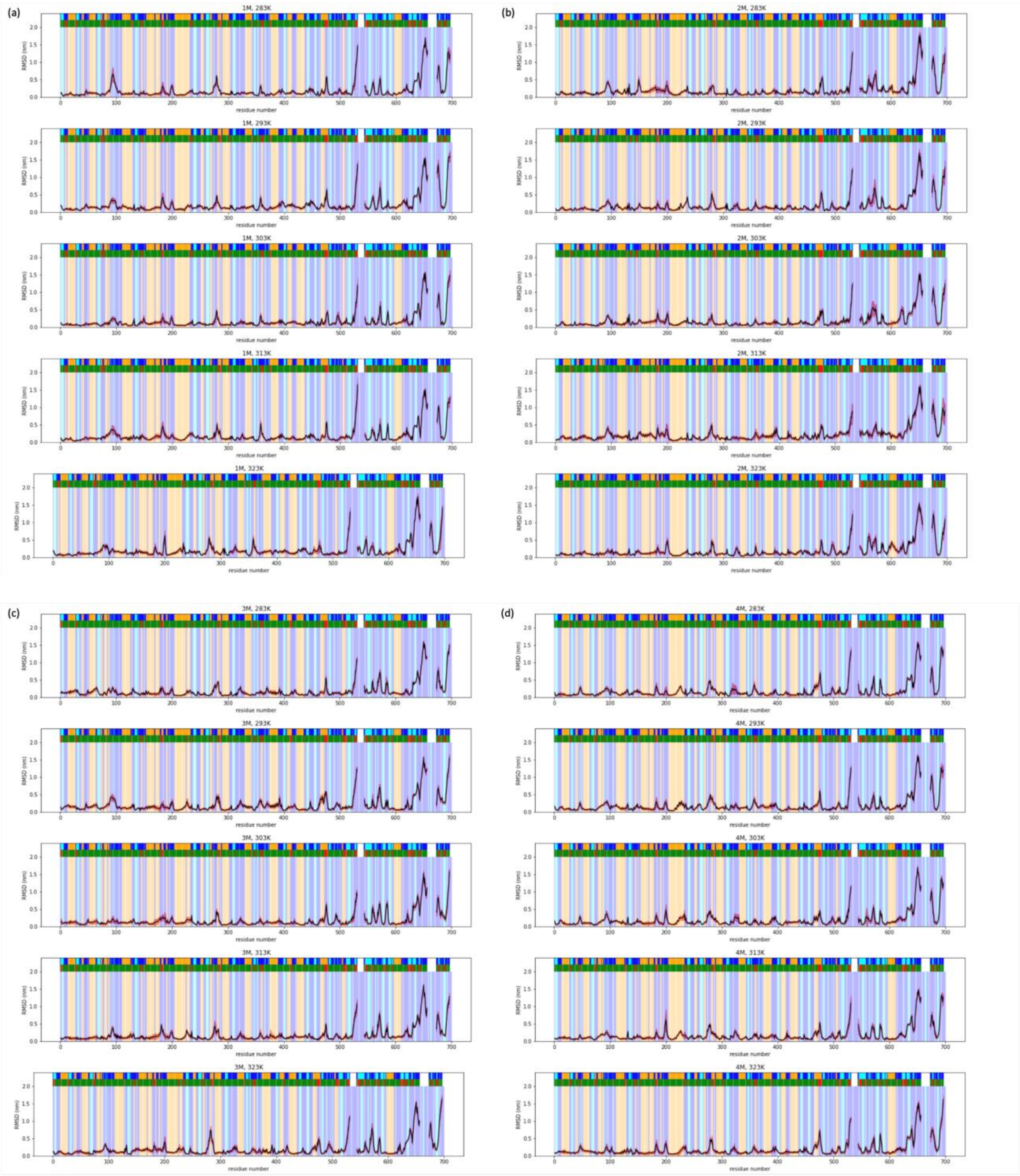

Figure S3. The average RMSD of backbone alpha carbons between the homology model and each condition is shown as the black line, with the red shaded outline showing the standard deviation of the average for each residue. The top row of cyan-orange-blue colors shows the secondary structure assignments for each residue in the crystal structure, and the faded cyan-orange-blue colors in the body of each graph shows the average secondary structure assignment for the residues in the homology model throughout the simulations. The secondary structure

assignments were made using MDTraj. A residue was assigned a certain secondary structure if the average frequency for that secondary structure was over 50%. Beta sheets are shown in cyan, alpha helices are shown in orange, and coils are shown in blue. The green and red colors in the row separating the secondary structure assignments of the crystal structure and homology model show the agreement between the two structures. If the crystal structure and homology model agree on the secondary structure assignment of a residue, then the agreement will be shown in green, but if they have different secondary assignments for a residue, then the disagreement will be shown in red. Parts of the crystal structure were missing and therefore are missing their secondary structure assignments and RMSD values. The conditions shown in (a) are 1M and 10 - 50 °C, (b) shows 1M and 10 - 50 °C, (c) shows 3M and 10 - 50 °C, and (d) shows 4M and 10 - 50 °C.

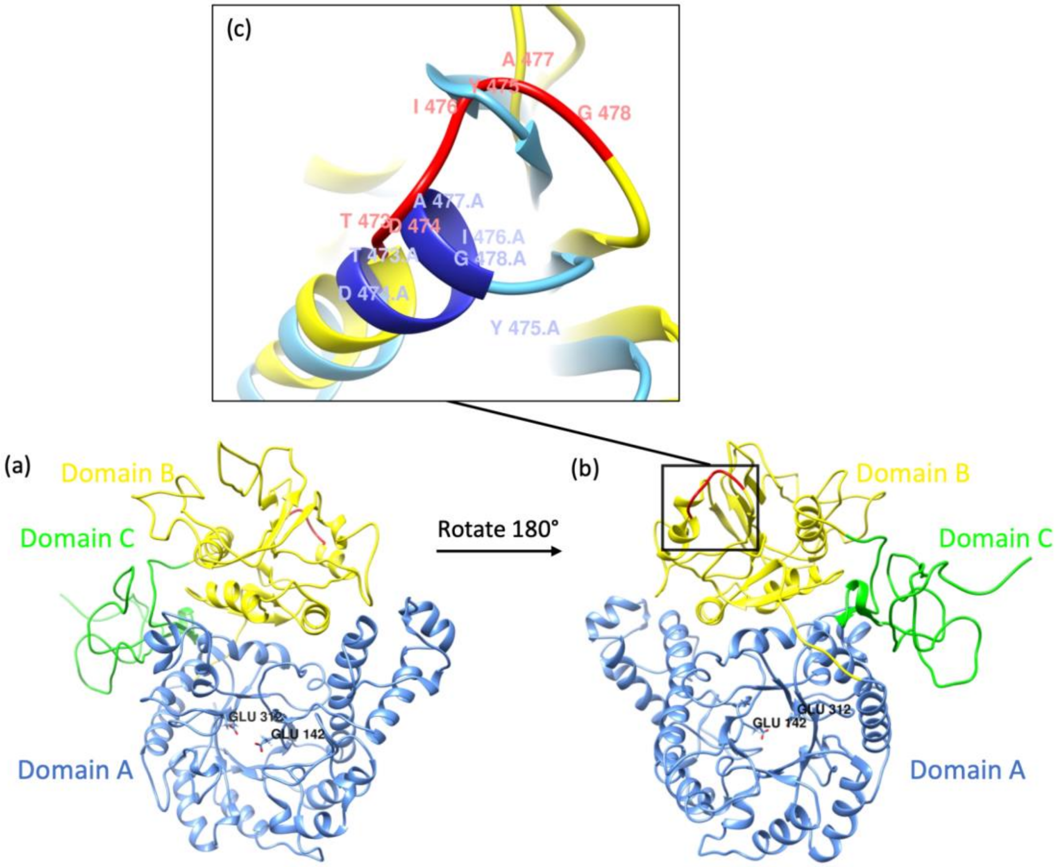

Figure S4. The largest secondary structure assignment disagreement (residues 473-478) between the homology model and the crystal structure is shown in red. The homology model is shown with the domains highlighted in different colors and the two catalytic residues labeled (GLU142 and GLU312) in (a) and rotated by 180 °C in (b). The crystal structure is superposed to the homology model and shown in blue in (c), with residues 473-478 colored in a dark blue.
